# Acute glycemic control in diabetics. How sweet is oprimal? Pro: Sweeter is better in diabetes

**DOI:** 10.1186/s40560-018-0336-2

**Published:** 2018-11-08

**Authors:** Rinaldo Bellomo

**Affiliations:** 1 Department of Intensive Care, Austin Hospital, The University of Melbourne, 145 Studley Rd., Heidelberg, Melbourne, 3084 Australia; 2 Australian and New Zealand Intensive Care Research Centre, School of Public Health and Preventive Medicine, Monash University, Prahran, Melbourne, Australia; 3 School of Medicine, University of Melbourne, Parkville, Melbourne, Australia; 4 Data Analysis Research and Evaluation (DARE) Centre, University of Melbourne, Parkville, Melbourne, Australia

**Keywords:** Glucose, Glycemia, Diabetes, Insulin, Randomized controlled trial, Hyperglycemia, Hypoglycemia

## Abstract

**Background:**

The optimal level of glycemic control in ICU patients has been the subject of intense investigation over the last 20 years. A pivotal study (the NICE-SUGAR study) involving more than 6,000 patients has established a target between 8 and 10 mmol/l (144 to 180 mg/dl) as the current standard of care. However, this study did not address whether patients with diabetes should be treated differently and, in particular, whether in such patients a higher glucose target should be used.

**Main concepts:**

The last decade has seen multiple studies aiming to describe the association between glycemia in mortality according to whether patients have or do not have diabetes and whether, if they have diabetes, pre-ICU admission glucose control (assessed by glycated hemoglobin A1c (HbA1c) levels) affects the relationship between acute glycemia and outcome. All such studies (now involving thousands and thousands of patients) have consistently shown that diabetic patients have a different relationship between acute glycemia and mortality. In particular, in diabetic patients, increasing glucose levels up to 15 mmol/l (270 mg/dl) or more are not associated with increased risk of death. In patients with a high HbA1c (> 7%) prior to ICU admission, targeting a glucose level below 10 mmol/l (180 mg/dl) is associated with increased risk compared with permissive hyperglycemia. Finally, a recent controlled study comparing a glucose target between 10 and 14 mmol/l (180 to 252 mg/dl) to a glucose target between 6 and 10 mmol/l (180 mg/dl) in diabetic patients found no advantage from tighter glycemia control. A randomized controlled study called LUCID is now underway to test the hypothesis that permissive hyperglycemia might be safer in diabetic patients admitted to the ICU.

**Conclusions:**

Until the results of the LUCID trial are available, the burden of evidence is in favour with targeting a more relaxed level of glycemia in diabetic patients (10–14 mmol/l; 180–252 mg/dl), especially in those with poor pre-admission glycemic control.

## Background

Current guidelines recommend insulin therapy to maintain a blood glucose level (BGL) below 10 mmol/l in all critically ill [1–3] and cardiac surgery patients [4]. These guidelines are supported by the findings of a pivotal trial: the Normoglycemia in Intensive Care Evaluation Survival Using Glucose Algorithm Regulation (NICE-SUGAR) trial. The NICE-SUGAR trial found that, in ICU patients, targeting a glucose of 8–10 mmol/l (144–180 mg/dl) compared with target glucose of 4.5–6.0 mmol/l (81–108 mg/dl) [5] reduced all-cause mortality at 90 days. However, in NICE-SUGAR, only limited information was available regarding premorbid glycemic control, diabetic status, and outcome. In particular, there was no information as to whether patients with diabetes would have a better outcome if their mean glycemia in the ICU was allowed to exceed 10 mmol/l (180 mg/dl). This issue is highly relevant to the approximately 25% of patients with diabetes who are currently admitted to ICUs in Western countries and who appear to be significantly different in terms of their relationship between acute glycemia and outcome.

## Main concepts

Patients with diabetes appear to respond differently to “conventional” glucose control compared with non-diabetic patients. For example, among type 2 diabetes patients with a glycated hemoglobin A1c (HbA1c) ≥ 7% (implying an average BGL of ≥ 8.5 mmol/l [153 mg/dl]; [6]), an average glucose level between 10 and 14 mmol/l (180–252 mg/dl) in the intensive care unit (ICU) was associated with lower mortality compared with recommended target levels [7]. Moreover, a large observational study found an association between diabetes and increased risk of hypoglycemia and mortality when conventional glucose control was applied in the ICU [8]. These observations suggest that a more liberal glycemic level (between 10 and 14 mmol/l) (180–252 mg/dl) in ICU patients with diabetes may both be safe and perhaps desirable [9–12]. Preliminary data support the notion that such “liberal” glycemic management may be particularly safe and desirable in those diabetic patients who have suboptimal pre-admission chronic glycemic control as shown by their HbA1c levels [10, 12]. However, until recently, there were limited controlled data to support these implications of observational studies. More recently, however, a controlled trial comparing conventional with liberal glycemic control in diabetic ICU patients was completed: the *S*afety of Gl*U*cose *E*levation *E*valuation *T*rial (SUEET) (ACTRN12615000216516) [13].

SUEET was a prospective, open-label, sequential, before-and-after study in ICU patients with diabetes. It aimed to compare the impact of liberal (10–14 mmol/l) (180–252 mg/dl) versus conventional (6 to 10 mmol/l) (198–180 mg/dl) glycemic control. SUEET compared 350 consecutive patients with diabetes who received liberal glucose control with a pre-intervention control population also of 350 consecutive patients with diabetes who received conventional glucose control.

SUEET found that liberal glycemic management decreased insulin therapy requirements and lowered glucose variability. It also found a trend towards decreased incidence of hypoglycemia with liberal glycemic control among those patients with HbA1c ≥ 7%. In addition, it found no difference in mortality (even after adjustment of baseline characteristics), duration of mechanical ventilation, or ICU-free days to day 30. Finally, it found no evidence of harm from liberal glycemic management in any patient subgroup.

SUEET also provided further insights into the consequences of liberal glycemic control in diabetic patients. For example, it found that almost half of glucose values spontaneously remained below 10 mmol/l (180 mg/dl) and that only a third of patients required insulin to maintain glucose levels within the target range. This observation implies that a liberal approach to glycemic control leads to no insulin-based intervention in 50% of diabetic ICU patients.

Furthermore, in keeping with the results of a pilot study of patients with a HbA1c ≥ 7%, where implementation of liberal glucose control was associated with half the risk of hypoglycemia, subgroup analysis of > 300 patients with a HbA1c ≥ 7% confirmed such benefits with a decrease in the incidence of hypoglycemia from 9.6 to 4.1%. Similarly, SUUET found that liberal control did not contribute to worsening kidney function in patients with diabetes. Moreover, the lack of a difference in the trajectory of white cell count during the first week in the ICU despite significant differences in glycemia suggests that concerns about increased risk of infection are unlikely to be correct.

Patients with diabetes reliably show no independent association between mean [14, 15] or peak glucose [16] and mortality. Among patients with HbA1c ≥ 7%, those with average blood glucose of 10–14 mmol/l (180–252 mg/dl) in the ICU appear more likely to survive to hospital discharge than those with a concentration in the conventional range [8]. Moreover, in patients with marked chronic hyperglycemia (HbA1c > 8.5%), acute hyperglycemia, and glycemic variability are not associated with increased mortality [16, 17]. Similarly, in a recent sequential period analysis of > 400 ICU patients with diabetes, Krinsley et al. assessed the impact of HbA1c-guided glycemic control on outcomes. These investigators delivered a “tighter” target in patients with HbA1c < 7% and a “looser” target in those with HbA1c ≥ 7%. Compared with their control period, such HbA1c-adjusted glycemic control significantly reduced the observed-to-expected mortality ratio [12]. Moreover, a large observational study of thousands of patients with diabetic ketoacidosis recently found that permissive hyperglycemia was associated with significantly greater survival compared with achieving a glucose level < 10 mmol/l in the first 24 h of ICU admission [18]. In their aggregate, these studies suggest that “permissive hyperglycemia” in ICU patients with diabetes, who are likely adapted to some degree of chronic hyperglycemia, is safe. Nonetheless, this field is evolving rapidly, with new interventions being considered [19], new technology being applied to glucose monitoring in the ICU [20], and the need to focus on long-term outcomes [21] all becoming new focuses of investigation.

## Conclusions

In summary, the above observations consistently suggest that liberal glucose control to a target between 10 and 14 mmol/l (180–252 mg/dl) decreases insulin administration requirements, lowers glucose variability, and reduces the risk of hypoglycemia, especially among those patients with poor pre-morbid glycemic control. Moreover, they suggest that insulin administration to maintain blood glucose within the conventional target range may be unnecessary in many critically ill patients with diabetes, irrespective of their premorbid glycemic control, illness severity, reason for ICU admission, and septic state. Finally, they justify the performance of a multicentre randomized controlled trial of liberal glycemic control in patients with diabetes. Such a trial (www.anzctr.org.au; ACTRN12616001135404) is now underway. Until the results of such trial (the *L*iberal Gl*U*cose **C**ontrol in Critically *I*ll Patients with Pre-existing Type 2 *D*iabetes trial—the LUCID trial) are available, the weight of evidence is clearly in favour of the concept that, in diabetic patients admitted to the ICU, sweeter is better.

## Abbreviation

Hba1c: Glycated hemoglobin A1c
